# QALYs without bias? Nonparametric correction of time trade‐off and standard gamble weights based on prospect theory

**DOI:** 10.1002/hec.3895

**Published:** 2019-06-24

**Authors:** Stefan A. Lipman, Werner B.F. Brouwer, Arthur E. Attema

**Affiliations:** ^1^ Erasmus School of Health Policy & Management (ESHPM) Erasmus University Rotterdam Rotterdam The Netherlands

**Keywords:** health state valuation, loss aversion, prospect theory, standard gamble, time trade‐off

## Abstract

Common health state valuation methodologies, such as standard gamble (SG) and time trade‐off (TTO), typically produce different weights for identical health states. We attempt to alleviate these differences by correcting the confounding influences modeled in prospect theory: loss aversion and probability weighting. Furthermore, we correct for nonlinear utility of life duration. In contrast to earlier attempts at correcting TTO and SG weights, we measure and correct all these tenets simultaneously, using newly developed nonparametric methodology. These corrections were applied to three less‐than‐perfect health states, measured with TTO and SG. We found considerable loss aversion and probability weighting for both gains and losses in life years, and we observe concave utility for gains and convex utility for losses in life years. After correction, the initially significant differences in weights between TTO and SG disappeared for all health states. Our findings suggest new opportunities to account for bias in health state valuations but also the need for further validation of resulting weights.

## 1 INTRODUCTION

In cost‐utility analyses (CUAs), incremental costs of medical technology are compared with incremental health benefits, commonly expressed in quality‐adjusted life years (QALYs). These QALYs (Pliskin, Shepard, & Weinstein, 1980) are obtained multiplying prospective life years by weights, sometimes referred to as “utilities.” QALY weights represent health‐related quality of life, such that 0 represents the subjective weight of the state “dead” and 1 that of full health. Several methods are used to obtain QALY weights, most notably standard gamble (SG) and time trade‐off (TTO). Empirical work, however, has demonstrated that QALY weights differ systematically between these two elicitation methods, with SG weights being higher than TTO weights (e.g., Bleichrodt & Johannesson, 1997; Torrance, 1976). As a consequence, QALY weights and, hence, outcomes of economic evaluations may depend on the health state valuation (HSV) method used.

Bleichrodt (2002) proposed that these discrepancies in elicited QALY weights may result from empirically invalid assumptions present in the theoretical frameworks underlying TTO and SG. More specifically, Bleichrodt argued that TTO and SG weights are biased as they are obtained under the assumptions of expected utility (EU) theory, which has been shown to be descriptively invalid for health outcomes (Bleichrodt, Abellan‐Perpiñan, Pinto‐Prades, & Mendez‐Martinez, 2007; Treadwell & Lenert, 1999). Additionally, although discounted QALY models exist (for an overview, see Hansen & Østerdal, 2006), TTO and/or SG weights are commonly derived under the linear QALY model, which assumes linear utility of life duration (and no discounting of future life years). However, many authors have found diminishing marginal utility of life years; that is, life years that occur in the distant future tend to receive less weight than do life years in the nearer future (Abellan‐Perpinan, Pinto‐Prades, Mendez‐Martinez, & Badia‐Llach, 2006; Bleichrodt & Pinto, 2005; Wakker & Deneffe, 1996). In order to obtain QALYs without bias, a methodological shift may be required in HSV towards the use of descriptive utility models such as prospect theory (PT).

PT is characterized by four tenets (Kahneman & Tversky, 1979; Tversky & Kahneman, 1992). These are (a) reference dependence—utility derived from a good is defined over differences from a reference point (RP), instead of over the overall consumption of that good; (b) loss aversion—the utility function has an inflection point at the RP and is steeper for losses than for gains; (c) diminishing sensitivity—utility is concave for gains and convex for losses, which indicates diminishing sensitivity to outcomes further from the RP; and (d) probability weighting—the decision maker overweighs small probabilities and underweighs large probabilities (Kahneman & Tversky, 1979; Tversky & Kahneman, 1992). PT is usually applied to decisions about money but has also been extended to health outcomes (Bleichrodt & Pinto, 2000; Miyamoto & Eraker, 1989). Importantly, as Bleichrodt (2002) proposed, the tenets modeled in PT will likely affect the TTO and SG methods differently, with loss aversion exerting an upward bias on both methods but utility curvature only affecting TTO whereas probability weighting only affects SG.

Given the increased importance of CUA in informing health policy (Drummond, Sculpher, Claxton, Stoddart, & Torrance, 2015), it is imperative to validly determine the weights that are ascribed to the relevant health states. The valuation of these health states, for example, when obtaining tariffs for the commonly used EuroQol (EQ‐5D) generic utility classification system (Versteegh et al., 2016), would necessarily occur within a descriptive context (Bleichrodt, Pinto, & Wakker, 2001). This means that the status quo of applying EU and/or the linear QALY model to derive TTO and SG weights (a) will not capture actual preferences, as these may include, for example, loss aversion, and (b) may lead to different TTO and SG weights according to Bleichrodt (2002). 1These statements hold regardless if one believes EU to be the normative standard (as Kahneman & Tversky, 1979, and Wakker, 2010, do), which would, for example, classify loss aversion as “irrational” or a bias. We will make no such claims and will refer to deviations of EU and the linear QALY model as generating bias in TTO and SG. As such, our main motivation is to address the discrepancy between TTO and SG weights by obtaining these QALY weights using derivations based on a descriptively valid but nonnormative theory (PT). We will refer to this process, where TTO and SG weights are obtained while incorporating loss aversion, nonlinear utility, and/or probability weighting into their derivation, as *correction for PT*. If correcting TTO and SG for PT is feasible, it could be used to correct observed responses in HSVs, allowing corrected weights to be used when calculating QALYs to express health benefits in CUAs, as commonly done.

Some studies have attempted to test Bleichrodt's (2002) predictions about PT and correct HSV techniques by assuming PT or adjusting for utility curvature (Attema & Brouwer, 2009; Martin, Glasziou, Simes, & Lumley, 2000; Oliver, 2003; van Osch, Wakker, van den Hout, & Stiggelbout, 2004; Wakker & Stiggelbout, 1995). Yet to date, no study has been able to simultaneously correct both TTO and SG for loss aversion, utility curvature, and probability weighting (see Appendix S1 for an overview of earlier studies on corrections). In this study, we adapted a recently proposed methodology (Abdellaoui, Bleichrodt, L'Haridon, & Van Dolder, 2016) to measure these three deviations without parametric assumptions and elicit TTO and SG weights without assuming EU or the linear QALY model. In other words, we provide the first empirical test of predictions by Bleichrodt (2002) and show how correcting for PT alleviates the discrepancies between TTO and SG.

Our study features several methodological improvements compared with previous attempts at correcting TTO and/or SG weights for PT (see Appendix S1). First, our adaptation of the nonparametric method (Abdellaoui et al., 2016) enables us to determine utility curvature, loss aversion, and probability weighting separately for each individual, without assuming a specific parameter or parametrical form for these functions (as opposed to work by van Osch et al., 2004, Martin et al., 2000, van der Pol & Roux, 2005). We believe this is relevant, as large heterogeneity typically exists for PT elicitations (Pinto‐Prades & Abellan‐Perpiñan, 2012), warranting an individual measurement approach. Furthermore, applying specific parametric forms within experimental elicitation can confound results (Abdellaoui, 2000), thus allowing considerable bias to remain after correction (Wakker, 2008; Wakker, 2010). Second, we attempt to append the heterogeneity surrounding RPs by providing all subjects with the same RP, which is a hypothetical expected life duration (following the successful procedure described in Attema, Brouwer, & L'Haridon, 2013). This is important, because even though reference dependence appears to be the most central tenet of PT, earlier work on the location of the RP suggests that individuals use multiple different health outcomes as RP (Bleichrodt et al., 2001; van Osch et al., 2004; van Osch & Stiggelbout, 2008; van Osch, van den Hout, & Stiggelbout, 2006).

## 2 THEORETICAL FRAMEWORK

We describe health outcomes as (*β*, *t*), where *β* represents health status and *t* indicates the age at which the health profile ends (e.g., living with chronic back pain until 70). Throughout, subscripts (e.g., *x* and *y*) are used to refer to possible health profiles faced by a single agent, with age of onset (e.g., current age) denoted by *t*_a_. We will often suppress *t*_a_ by denoting (*β*_*x*_, *t*_*x*_) as (*β*_*x*_, *T*_*x*_), with duration defined by *T*_*x*_ = *t*_*x*_ − *t*_a_ ≥ 0. We refer to (*β*_*x*_, *T*_*x*_) as chronic health profiles. We let (*β*_*x*_, *T*_*x*_)_*p*_(*β*_*y*_, *T*_*y*_) denote the risky prospect that provides health profile (*β*_*x*_, *T*_*x*_) with probability *p* and health profile (*β*_*y*_, *T*_*y*_) with probability 1 − *p*. Preferences are denoted using the conventional notations ≻, ≽, and ∽ to represent strict preference, weak preference, and indifference, respectively. Also, we assume weak‐ordered preferences; that is, they are complete, meaning that decision makers have preferences over risky prospects, and transitive (if *x* ≽ *y* and *y* ≽ *z*, then *x* ≽ *z*). Health profiles (*β*_*x*_, *T*_*x*_) starting and ending at *t*_a_ (so that *t*_a_ = *t*_*x*_) will thus have *T*_*x*_ = 0 (i.e., they equal immediate death), and, for brevity, we will denote such profiles of the form (*β*_*x*_, 0) as *D*, for any *β*_*x*_. As in Miyamoto, Wakker, Bleichrodt, and Peters (1998), we assume indifference between all profiles denoted *D* for any *β*. Finally, we assume monotonicity for duration, that is, (*β*_*x*_, *T*_*x*_) ≻ (*β*_*x*_, *T*_*y*_) for *T*_*x*_ > *T*_*y*_ and any *β*_*x*_.

The general QALY model assumes that preferences for health profiles (*β*_*x*_, *T*_*x*_) are represented by the general utility function *V*(*β*_*x*_, *T*_*x*_) = *U*(*β*_*x*_) * *L*(*T*_*x*_). In this model, *L*(*T*) and *U*(*β*) denote utility functions over life years or health status, respectively. This QALY model, and the preference foundations underlying it, typically relies on EU to some extent (for axiomatizations, see Miyamoto & Eraker, 1989, Miyamoto & Eraker, 1988). To derive corrected TTO and SG weights, we will extend this model to incorporate insights from PT under risk. That is, we assume that preferences can be represented by the general QALY model, including the extensions we outline below.

Several preliminaries are required before defining our full model (Equations (1) and 2). We assume that preferences for health profiles are defined relative to an RP, which we denote as (*β*_*r*_, *T*_*r*_). Following Wakker (2010), we define this RP as a point of comparison, which may differ during different parts of the analysis. Given that no plausible theory of RP selection is available (Wakker, 2010), we let the RP depend on framing of the decision context. Hence, (*β*_*r*_, *T*_*r*_) refers to an expected health profile described in a decision task, which is taken as the neutral point. This health profile has health status *β*_*r*_, endured for *T*_*r*_ years. Throughout, for brevity, we denote the duration of all other health profiles as deviations from the RP; that is, we denote health profiles (*β*_*x*_, *T*_*x*_) as (*β*_*x*_, *T*_x_^*^) with *T*_x_^*^ = *T*_*x*_ − *T*_*r*_ in *β*_*x*_. We will restrict our model to health profiles (*β*_*x*_, *T*_x_^*^) ≽ *D* with *β*_*x*_ ≽ *β*_*r*_ for any $\;T_{x}^{*}$ . In other words, we assume our model holds for a restricted outcome domain including only health profiles weakly preferred to immediate death, where health status remains at *β*_*r*_ or is improved.

Within this outcome domain, we model PT by incorporating sign dependence for life duration, that is, by modifying *L*(*T*) in the general QALY model to *L* ^*i*^(*T* ^*^). In our model, *L* ^*i*^(*T* ^*^) is a standard, real‐valued ratio scale utility function with *L* ^+^(*T*_*r*_) = 0, which may be different for gain outcomes ( $\beta_{x},T_{x}^{*},$ with *β*_*x*_ ~ *β*_*r*_ and $T_{x}^{*}\geq 0)$ and loss outcomes ( $\beta_{x},T_{x}^{*},$with *β*_*x*_~*β*_*r*_ and $T_{x}^{*}<0$). We do not modify *U*(*β*) in our model, which implies that changes in health status will be evaluated as in the conventional general QALY model. We incorporate loss aversion 2In our simplified approach, we model PT over life duration by assuming attribute‐specific evaluation (as in Bleichrodt et al., 2009). Loss aversion is, thus, defined over life duration, as it is not meaningful on *U*(*β*_*x*_) when health status is considered a qualitative measure (Bleichrodt and Miyamoto, 2003). This does not affect our analysis, as we only consider improvements in health status. by taking *L* ^−^(*T* ^*^) = *λL* ^*i*^(*T* ^*^) for *T* ^*^ < 0. Here, *λ* denotes a loss aversion index, with *λ* > 1 (*λ* = 1, *λ* < 1) indicating loss aversion (loss neutrality, gain seeking). Furthermore, we incorporate nonlinear weighting of probabilities by incorporating probability weighting functions *w* ^*i*^(*p*), *i* = +, −, for gains and losses respectively, that assign a number to each probability *p*, with *w* ^*i*^(0) = 0 and *w* ^*i*^(1) = 1.

We will apply this model to risky prospects with at most two outcomes, that is, binary prospects. Thus, preferences over risky prospects with both gain and loss outcomes, that is, ${\left(\beta_{x},T_{x}^{*}\right)}_{p}\left(\beta_{y},T_{y}^{*}\right)$, with $T_{x}^{*}\geq 0>T_{y}^{*}$ are evaluated by

(1) $$w^{+}\left(p\right)U\left(\beta_{x}\right)L^{+}\left(T_{x}^{*}\right)+w^{-}\left(1-p\right)U\left(\beta_{y}\right)L^{-}\left(T_{y}^{*}\right),$$

whereas preferences over risky prospects $\;{\left(\beta_{x},T_{x}^{*}\right)}_{p}\left(\beta_{y},T_{y}^{*}\right)$ for either gains or losses are evaluated by

(2) $$w^{i}\left(p\right)U\left(\beta_{x}\right)L^{i}\left(T_{x}^{*}\right)+\left(1-w^{i}\left(p\right)\right)U\left(\beta_{y}\right)L^{i}\left(T_{y}^{*}\right),i=+,-,$$

where *i* = + [−] when $\;T_{x}^{*},T_{y}^{*}>\left[<\right]\;0$, that is, both outcomes are gains or losses. Whenever *w* ^*i*^(*p*) = *p*, *λ* = 1, and no distinction is made between gains and losses (i.e., no reference dependence), our model reduces to the general QALY model.

### 2.1 SG and TTO correction for PT

TTO weights are obtained by eliciting duration *T*_*y*_, which yields indifference between (*β*_*x*_, *T*_*x*_) and (*FH*, *T*_*y*_), with *T*_*x*_ > *T*_*y*_. SG weights, on the other hand, are obtained from indifferences between a certain outcome (*β*_*x*_, *T*_*x*_), and a risky prospect (*FH*, *T*_*x*_)_*p*_(*D*), where *p* is normally varied until indifference is obtained. Often, TTO and SG weights (i.e., *U*(*β*_*x*_)) are derived under the assumptions of EU and the linear QALY model, which is a special case of the general QALY model with *L*(*T*) = *T*, *U*(*FH*) = 1, and *V*(*D*) = 0. Under these assumptions, indifferences (*β*_*x*_, *T*_*x*_) ~ (*FH*, *T*_*y*_) and (*β*_*x*_, *T*_*x*_) ~ (*FH*, *T*_*x*_)_*p*_(*D*) allow derivation of TTO and SG weights for health state *β*_*x*_ by $U\left(\beta_{x}\right)=\frac{T_{y}}{T_{x}}$ and U(*β*_*x*_) = *p*, respectively.

Our correction for PT involves deriving TTO and SG weights by means of our theoretical model based on PT. The application of our theoretical model requires assumptions about the RP used in TTO and SG. Typically, TTO and SG exercises are framed with the impaired health state (*β*_*x*_, *T*_*x*_) as RP. Furthermore, earlier work on SG 3No empirical work exists studying the RP for TTO. Here, we assumed that it coincides with that of SG and with how TTO is typically framed. If the time spent in perfect health (i.e., *FH*, *T*_*y*_) is taken as RP instead, Equation 3 cannot be applied. This also holds for SG; that is, Equation 4 is only valid if the RP is actually (*β*_*x*_, *T*_*x*_). has suggested that the outcome that remains constant, that is, the time spent with reduced health status (*β*_*x*_, *T*_*x*_), usually is taken as RP (Bleichrodt et al., 2001; van Osch et al., 2006). Hence, throughout the paper, we will make the following assumption about the RP for TTO and SG: (*β*_*r*_, *T*_*r*_) = (*β*_*x*_, *T*_*x*_).

Under these assumptions, TTO indifferences (*β*_*x*_, *T*_*x*_) ~ (*FH*, *T*_*y*_) allow the following derivation for *U*(*β*_*x*_): 4Equations 3 and (4) apply a scaling of *L* ^*i*^(*T* ^*^), where the utility of the lowest outcome is set to −1, for simplicity (i.e., *L* ^−^(*T*_*a*_) = −1). For elaborate proofs of Equations 3 and (4) under our theoretical model, see Appendix S2.

(3) $$U\left(\beta_{x}\right)=\frac{L^{-}\left(T_{y}^{*}\right)+1}{\left(1-\lambda \right)L^{-}\left(T_{y}^{*}\right)+1},$$

whereas SG indifference (*β*, *T*_*x*_) ~ (*FH*, *T*_*x*_)_*p*_(*D*) allows the following derivation for *U*(*β*_*x*_) as in Bleichrodt et al. (2001):

(4) $$U\left(\beta_{x}\right)=\frac{w^{+}\left(p\right)}{w^{+}\left(p\right)+\lambda w^{-}\left(1-p\right)}.$$

### 2.2 Parameter elicitation

In order to correct both TTO and SG weights for PT, that is, to be able to compute the outcome of Equations (3) and (4), one needs to elicit the following: (a) *L* ^*i*^(*T* ^*^) with $T_{x}^{*}$ as RP to allow estimation of $L^{-}\left(T_{y}^{*}\right)$, (b) probability weighting functions *w* ^*i*^(*p*), *i* = +, −, and (c) a loss aversion coefficient *λ*, which reflects overweighting of losses with $T_{x}^{*}\;$as RP. This means that *t*_*x*_ should be kept constant across TTO and SG and the elicitation of *L* ^*i*^(*T* ^*^), to ensure that *λ* refers to the same theoretical construct throughout (i.e., the same kink around the RP, see Section 4.4).

## 3 METHODS

We report the results of an experiment in which we compare TTO and SG weights derived assuming EU and the linear QALY model to QALY weights corrected for PT (i.e., by Equations (3) and 4). In this experiment, PT parameters were elicited using methodology based on the work by Abdellaoui et al. (2016). To reduce the influence of order effects and test for consistency, multiple counterbalancing procedures were conducted between participants and consistency checks were in place (see Appendix S3). The experiment was computerized in Matlab. Subjects were 99 students of the Rotterdam School of Management (58 female) who were rewarded course credits. Experimental sessions lasted for approximately 55 min and were run on computers in sessions of four subjects sitting adjacently in separate cubicles. An instructor was present at all times to answer questions.

### 3.1 TTO and SG weight elicitation

We elicited TTO and SG weights for a total of four health states (one practice state) from the EQ‐5D‐5L (five level) descriptive system (Herdman et al., 2011). These health states reflected an array of mildly aversive health states, in order to avoid health states that could be considered worse than death (Dolan, 1997). The following health states were used: 22222 (practice, *β*_*p*_), *β*_1_ = 21211, *β*_2_ = 31221, and *β*_3_ = 32341. We applied a bisection choice‐based elicitation procedure with four consecutive choices, as choice‐based procedures produce more consistent measurements than matching (Noussair, Robin, & Ruffieux, 2004). Subjects were asked to imagine having lived until age 50 in perfect health after which they contracted a disease that would affect their quality of life for their remaining life expectancy of 20 years. TTO and SG were completed for these remaining 20 years (i.e., *t*_a_ = 50). In both cases, the maximum expected age of death was 70 years; that is, subjects made decisions with regard to the quality of life for age 50 to 70 (followed by death), which ensured that *t*_*x*_ was constant for both TTO and SG.

### 3.2 Nonparametric method

We adapted Abdellaoui et al.'s (2016) nonparametric methodology to measure PT under risk in the health domain. In order to elicit *L* ^*i*^(*T* ^*^) with the same *t*_*x*_ as RP as in TTO and SG, we instructed subjects to take living from current age until 70 in perfect health as RP, that is, (*β*_*r*_, *T*_*r*_) = (*FH*, 70 − *t*_a_). Elicitation consisted of four stages (an elaborate description of the method and instructions can be found in Appendices S1, S4, and S5). The first stage connected utility for gains (*L* ^+^(*T* ^*^)) to the utility for losses (*L* ^−^(*T* ^*^)). The second and third stages employed the trade‐off method of Wakker and Deneffe (1996) to measure a standard sequence of utility for gains and utility for losses, respectively. The fourth stage measured probability weighting, separately for gains and losses; that is, *w* ^+^(*p*) and *w* ^−^(*p*). Our methodology thus makes it possible to completely elucidate PT's tenets in the health domain, without imposing parametric assumptions on *L* ^*i*^(*T* ^*^) and *w* ^*i*^(*p*). Each of the four stages had slightly different instructions (see Appendix S5), providing the context for the trade‐offs that subjects were required to make. Subjects had to choose between two medicines that could amend their situation but would not affect their life expectancy, which remained constant at perfect health. All indifferences were elicited using a bisection choice‐based procedure with a slider (following Abdellaoui et al., 2016) where subjects first performed three binary choices. This procedure zoomed in to the point at which subjects would become indifferent but still allowed subjects to specify the final value and adjust accordingly. To allow estimation of $L^{-}\left(T_{y}^{*}\right)$ in Equation (3) regardless of the amount of years given up in TTO, subjects' standard sequence continued to at least 20 years above and below *t*_*x*_ (i.e., living until 70), to avoid extrapolation beyond the measured curve 5After 25 steps, the standard sequence elicitation was terminated to avoid overburdening our subjects. When necessary, $L^{-}\left(T_{y}^{*}\right)$ was obtained by extrapolation..

### 3.3 Analyses of curvature for L ^i^(T)

We used two methods to investigate the curvature of *L* ^*i*^(*T* ^*^), that is, utility curvature: a nonparametric method and a parametric method (similar to Abdellaoui et al., 2016). For these analyses of utility curvature, we normalized all durations by dividing through subjects' highest absolute elicited duration for gains and losses, respectively ( $T_{\mathrm{kG}}^{*}$ or $-T_{\mathrm{kL}}^{*})$. This resulted in *T* ^*^ being in the range [−1, 1]. Next, we calculated the area under the curve (AUC) of *L* ^*i*^(*T* ^*^) separately for both domains, by setting $L^{+}\left(T_{\mathrm{kG}}^{*}\right)=1$ and $L^{-}\left(T_{\mathrm{kL}}^{*}\right)=-1$. If utility of life duration is linear, the area under this normalized curve equals one half. Utility for gains in life duration is convex (concave) if the AUC is smaller (larger) than one half, whereas for losses, the opposite direction holds (convex > ½, concave < ½). This method of analyzing utility curvature is nonparametric. We also analyzed *L* ^*i*^(*T* ^*^) parametrically by employing the most commonly used power utility family using nonlinear least squares, using the same normalizations. For this family, *L* ^+^(*T* ^*^) = (*T* ^*^)^*α*^ and *L* ^−^(*T* ^*^) = −(−(*T* ^*^)^*α*^) with *α* > 0. For gains [losses], *α* > 1 corresponds to convex [concave] utility, *α* = 1 corresponds to linear utility, and *α* < 1 corresponds to concave [convex] utility.

### 3.4 Analyses of loss aversion

Several definitions of loss aversion exist, with *λ* being interpreted in various manners (see Köbberling & Wakker, 2005). Köbberling and Wakker (2005) defined loss aversion (*λ*) as the kink of utility at the RP. That is, they define loss aversion as $U_{↑}^{\prime }\left(0\right)/U_{↓}^{\prime }\left(0\right)$, with $U_{↑}^{\prime }\left(0\right)$ representing the left derivative and $U_{↓}^{\prime }\left(0\right)$ the right derivative of *U* at the RP. Hence, we computed each subject's coefficient of loss aversion (*λ*) over the first steps in their standard sequence for gains and losses, denoted as $x_{1}^{+}\;$and $x_{1}^{-}$. Loss aversion is then defined as the ratio of $L^{-}\left(x_{1}^{-}\right)/x_{1}^{-}$ over $L^{+}\left(x_{1}^{+}\right)/x_{1}^{+}$, which is equal to $x_{1}^{+}/-x_{1}^{-}$ (Abdellaoui et al., 2016). A subject was classified as loss averse if $x_{1}^{+}/-x_{1}^{-}$ > 1, loss neutral if $x_{1}^{+}/-x_{1}^{-}$ = 1, and gain seeking if $x_{1}^{+}/-x_{1}^{-}$ < 1 (as in Wakker, 2010).

### 3.5 Probability weighting

We used certainty equivalences using varying probabilities to elicit the weighting functions, similar to Attema, Bleichrodt, and L'haridon (2018). In particular, we used linear interpolation to obtain a *w* ^+^(*p*) and *w* ^−^(*p*), using *p* = 0.1, 0.3, 0.5, 0.7, 0.9. Furthermore, we used Tversky and Kahneman's one‐parameter inverse S‐shaped probability weighting function *w*^*i*^(*p*) = *p*^*γ*^/(*p*^*γ*^+(1 − *p*)^*γ*^)^1/*γ*^ with *i* = +, −, estimated by nonlinear least squares. The *γ*‐parameter controls for the shape of the probability weighting function. If *γ* = 1, there is no probability transformation and *w* ^*i*^(*p*) = *p*. However, if *γ* < 1, decision makers underweight large probabilities and overweight small probabilities. This corresponds to the commonly found inverse S‐shaped weighting function. If *γ* > 1, the opposite pattern holds, corresponding to an S‐shaped weighting function.

## 4 RESULTS

Two subjects expressed unwillingness to trade off any life years, which caused the experiment to fail. These subjects were removed from further analyses. As can be seen in Appendix S3, we included several repetitions to test for consistency. At the aggregate level, we observed significant differences between the consistency indifference value and the value for $x_{2}^{i}$ (i.e., the second step) in the standard sequence elicitation for both gains and losses (paired *t* tests: *p*s < .01). Furthermore, we found a difference for the consistency checks in the probability sequence for gains (paired *t* test: *p*s = .007), but not for losses (paired *t* tests: *p*s = .62). Correlations between consistency checks and original values were high, suggesting strong association between these values (Kendall's *τ*s > 0.51, *p*s < .003).

Twenty‐nine subjects violated monotonicity for health states, which indicates that they valued at least one health state, which was better or equal on each dimension lower than their dominated counterpart (e.g., 21211 vs. 31221). As we consider that it is plausible that all subjects prefer more health to less, we reran the full analyses excluding these subjects and found no differences in the main results. Hence, we report the results for the full sample (*n* = 97).

### 4.1 Curvature of L ^+^(T) and L ^−^(T)

We observed median AUC for gains equal to 0.555, and for losses, this nonparametric analysis produced a median AUC of 0.561, which were both significantly different from 0.5 (Wilcoxon signed ranks tests: *ps* < .001). After parametrically fitting a power function to the data, we found a median *α* of 0.787 for gains and 0.757 for losses (significantly smaller than 1, Wilcoxon signed ranks tests: *ps* < .001). Thus, both parametric and nonparametric results demonstrated *L* ^+^(*T* ^*^) to be concave and *L* ^−^(*T* ^*^) to be convex.

Table 1 shows the classification of subjects' curvature for gains (*L* ^+^(*T* ^*^)) and losses (*L* ^−^(*T* ^*^)) at the individual level, both parametrically and nonparametrically. The most common pattern was concave curvature for *L* ^+^(*T* ^*^) and convex curvature for *L* ^−^(*T* ^*^) as was found in an earlier implementation of this method (Attema et al., 2018). This conclusion holds for both nonparametric (53%) and parametric (53%) results.

**Table 1:** Classification for curvature of L ^+^(T ^*^) and L ^−^(T ^*^) at the individual level

Gains L ^+^(T ^*^)	Losses—L ^−^(T ^*^)			
	Concave	Convex	Linear	Total
Nonparametric				
Concave	19	51	0	70
Convex	7	17	1	25
Linear	0	1	1	2
Parametric				
Concave	19	51	0	70
Convex	6	18	1	25
Linear	0	1	1	2

### 4.2 Loss aversion

Utilizing Köbberling and Wakker's (2005) definition, we found a median loss aversion index of *λ* = 2 (interquartile range: 1.00–3.52). Thus, we found considerable loss aversion at the aggregate level, with the median being significantly higher than 1 (Wilcoxon test: *p* < .001). At the individual level, the majority of subjects demonstrated loss aversion, with 72% (*n* = 70) classifying as loss averse, and 15% (*n* = 15) and 13% (*n* = 12) classifying as loss neutral or gain seeking, respectively.

### 4.3 Probability weighting (w ^i^(p))

**Figure 1:**
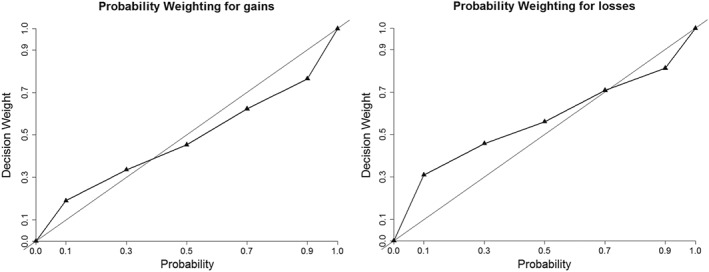
Probability weighting functions for gains (w ^+^(p)) and losses (w ^−^(p))

Figure 1 shows the median decision weights assigned to *p* = 0.1, 0.3, 0.5, 0.7, 0.9. As can been seen from the plots, we observe inverse S‐shaped probability weighting for both gains and losses, with more pronounced overweighting of small probabilities for losses. Using Tversky and Kahneman's one‐parameter function, we found a median *γ* = 0.92 for gains and a median *γ* = 0.84 for losses (both significantly lower than 1, Wilcoxon tests: *p*s < .04). Both analyses demonstrated that the typical inverse S‐shaped probability transformation was the most prevalent in our data, for both gains and losses. Moving to the individual level, for gains, we found *γ* < 1 for 56 subjects (58%) and *γ* > 1 for 41 subjects (42%). For losses, we found more pronounced inverse S‐shaped probability weighting, with 71 (73%) and 26 (27%), respectively.

### 4.4 Health state correction

Table 2 shows QALY weights for all health states elicited using TTO and SG, where uncorrected refers to weights elicited assuming EU and linear QALYs, whereas corrected weights are elicited by means of Equations (3) and (4). To test the sensitivity of our results to linear interpolation, we also corrected TTO and SG weights by using power utility to estimate $L^{-}\left(T_{y}^{*}\right)$ and the Kahneman and Tversky probability weighting function to estimate *w* ^+^(*p*) and *w* ^−^(1 − *p*); these are indicated by “Parametric Corrections” in Table 2. An initial difference in TTO and SG weights existed (paired *t* tests, all *p*s < .001), with SG weights being higher than TTO for all *β*_*x*_. Our results show that the corrected weights were lower than the uncorrected weights for TTO and SG (paired *t* tests: all *p*s < .01). The initially significant difference between the uncorrected weights only disappeared for all *β* after applying nonparametric corrections (paired *t* tests: all *p*s > .09). The parametric corrections left significant and substantial differences between TTO and SG weights.

**Table 2:** Overview of mean weights [standard deviation] for health states β_1–3_ for TTO and SG including differences between methodologies under multiple corrections

Correction	Health state	TTO weight	SD	SG weight	SD	Difference
Uncorrected	*β*_1_: 21211	0.665	[0.268]	0.75	[0.25]	−0.085*
	*β*_2_: 31221	0.605	[0.259]	0.706	[0.261]	−0.101*
	*β*_3_: 32341	0.39	[0.259]	0.518	[0.276]	−0.128*
Nonparametric	*β*_1_: 21211	0.492	[0.331]	0.506	[0.295]	−0.014 ns
	*β*_2_: 31221	0.442	[0.313]	0.456	[0.287]	−0.014 ns
	*β*_3_: 32341	0.279	[0.27]	0.319	[0.229]	−0.039 ns
Parametric	*β*_1_: 21211	0.496	[0.325]	0.598	[0.319]	−0.102*
	*β*_2_: 31221	0.449	[0.307]	0.558	[0.322]	−0.109*
	*β*_3_: 32341	0.295	[0.272]	0.387	[0.303]	−0.092*

Abbreviations: SG, standard gamble; TTO, time trade‐off.

* Differences were significant at *p* < .001 for paired *t* tests.

Finally, we performed four isolated corrections. For the sake of brevity, we only report the results of the nonparametric corrections (see the Supporting Information for results of these analyses for parametric corrections). First, we corrected TTO for utility curvature only, with *λ* = 1. Second, TTO weights were corrected for loss aversion only, with linear utility (i.e., *L* ^*i*^(*T* ^*^) = *T* ^*^). Third, we corrected SG for probability weighting only, with *λ* = 1. Finally, SG weights were corrected for loss aversion only, with *w* ^*i*^(*p*) = *p*. This allows us to demonstrate the influence of each correction in isolation. Table 3 shows that correcting for loss aversion had a stronger downward influence on TTO weights than correcting for curvature of *L* ^*i*^(*T* ^*^), and both correcting for probability weighting and correcting for loss aversion had a substantial negative influence on SG weights.

**Table 3:** Isolated effects of corrections for UC, LA, and PW for TTO and SG weights [standard deviation in brackets]

Health state	Uncorrected weight		UC only		LA only		PW only	
TTO: Implication	*λ* = 1 and *L* ^*i*^(*T* ^*^) = *T* ^*^		*λ* = 1		*L*(*T* ^*^) = *T* ^*^			
*β*_1_: 21211	0.665	[0.268]	0.611	[0.296]	0.537	[0.311]		
*β*_2_: 31221	0.605	[0.259]	0.558	[0.287]	0.474	[0.3]		
*β*_3_: 32341	0.39	[0.259]	0.364	[0.278]	0.288	[0.259]		
SG: Implication	*λ* = 1 and *w* ^*i*^(*p*) = *p*				*w* ^*i*^(*p*) = *p*		*λ* = 1	
*β*_1_: 21211	0.75	[0.25]			0.63	[0.307]	0.643	[0.246]
*β*_2_: 31221	0.706	[0.261]			0.584	[0.305]	0.597	[0.249]
*β*_3_: 32341	0.518	[0.276]			0.387	[0.278]	0.459	[0.218]

Abbreviations: LA, loss aversion; PW, probability weighting; SG, standard gamble; TTO, time trade‐off; UC, utility curvature.

## 5 DISCUSSION

This paper provides the first empirical test of Bleichrodt's (2002) predictions about PT, demonstrating that it may be possible to correct the weights typically used in HSV, that is, to reduce bias in TTO and SG.

We estimated the full set of PT's parameters in the health domain, in order to obtain more descriptively valid outcomes, which can be used in the QALY model. Our results are consistent with PT (Kahneman & Tversky, 1979): We observe concave utility curvature for gains and convex utility curvature for losses, inverse S‐shaped probability weighting, and considerable loss aversion. In general, the estimates of utility curvature for gains in life duration and loss aversion (when applicable) of earlier work are similar to ours (e.g., Attema, Brouwer, & L'Haridon, 2013; Bleichrodt & Pinto, 2000; Bleichrodt & Pinto, 2005), but different results are found for the utility function for losses in life duration. These differences might be explained by methodological differences, which is a hypothesis that could be tested in future work. Furthermore, we replicated the typical finding that SG weights are higher than TTO weights. By means of corrections similar to those proposed by Bleichrodt et al. (2001), we attempted to remove the systematic bias in these weights, by simultaneously accounting for loss aversion, probability weighting, and utility curvature. Consequently, as predicted by Bleichrodt (2002), the weights assigned to both TTO and SG were markedly lower than their uncorrected counterparts. Moreover, they were no longer significantly different.

Although successful attempts at correcting SG and/or TTO weights using parametric methodology are reported in earlier work (Martin et al., 2000; van der Pol & Roux, 2005; van Osch et al., 2004), our parametric corrections were not able to fully account for the discrepancies between these methods. This seemed to be driven by SG weights remaining higher when parametric estimations for probability weighting were used. Given that our nonparametric estimations of probability weighting allowed full flexibility of the weighting function (see Abdellaoui, 2000), these findings suggest that parametric estimations of probability weighting may produce different results.

Our results demonstrate that, considered in isolation, loss aversion had a stronger downward influence on TTO weights than utility curvature, whereas both probability weighting and loss aversion lowered SG weights considerably. Although these findings are generally in line with previous studies, we observed a downward effect of correcting TTO for utility curvature. Probably, this is caused by the convexity found for losses in life years and the framing of our TTO and SG exercises (which both featured losses in life years from the RP in a reduced health state). Future work could shed light on the degree to which this discrepancy may be caused by the nonparametric method or the framing used in our work.

Several limitations of our study need noting. First, several subjects violated monotonicity for the health states used. Although excluding these subjects from the sample did not alter our results, we expect that these errors in decision making are to be attributed to either (a) imprecision of preferences or (b) error propagation, that is, early errors cascading into later stages of the task. Considering the use of only relatively mild health states, for which subjects may have no precise preference ordering in mind, some overlap may occur within our method. Regarding error propagation, it is good to note that during utility elicitation, subjects could rectify errors by adjusting the final indifference value on the slider to any nondominant value in life years, that is, fix their earlier “errors.” Testing for error propagation, by performing an error simulation as described by Bleichrodt and Pinto (2000), confirmed that errors did not have a propagating effect on the standard sequence we elicited for gains and losses. 6The difference between TTO and SG weights not was not significant in all simulations (*k* = 1,000) for *β*_1_ and *β*_2_, while replicating our results in the majority of simulations for *β*_3_ (over 70%). These simulations suggest that our correction method is quite robust to error propagation.


Second, concerns may be raised about the role of the RP in this paper. We find that the observed discrepancies between TTO and SG can be removed by correcting under the assumption that decision makers utilize the guaranteed outcome (*β*_*x*_, *T*_*x*_) as RP (which ensures that *t*_*x*_ remains constant). However, earlier work on health‐related preferences has suggested that individuals may also use their own current health and life expectancy as RP (van Nooten & Brouwer, 2004; van Nooten, Koolman, & Brouwer, 2009). In our work, we found no evidence of such effects. 7We tested for associations between subjects' self‐reported life expectancy and their estimates for loss aversion, utility curvature, and probability weighting; no such associations were observed for raw and corrected health state weights (all Kendall's *τ*s < 1.52, all *p*s > .13). A related limitation concerns our assumption that subjects use the fixed outcome in both TTO and SG as their RP, which is crucial for our results as our corrections depend on a constant *T*_*r*_ throughout the multiple parts of the experiment. Earlier work, however, demonstrated that SG subjects may also use the time spent in full health as their RP (van Osch & Stiggelbout, 2008). To our knowledge, such work does not exist for TTO methods. Therefore, future work should explore the possibility of correcting under the assumption that subjects use full health as RP, for both TTO and SG.

Finally and perhaps most importantly, the primary goal of the present research was merely to provide the first empirical test of Bleichrodt's (2002) predictions for TTO and SG weights, and our findings should be interpreted in this context. We observed considerable differences to nationally representative findings. For example, the Dutch tariff (Versteegh et al., 2016) for health state *β*_1_ (21211) is 0.876, whereas we elicited a raw TTO weight of 0.665. Our sample, consisting of young, healthy students will have contributed strongly to this initial discrepancy, next to differences in methodology. We also note that after correction, the discrepancy between tariffs and corrected weighs increases. After the nonparametric correction, the QALY value of state *β*_1_ decreases to 0.492. Clearly, this calls for further investigation of the methods used here, also in other (general public) samples, in order to further explore the impact of corrections and further refine the methods used. This future research may also clarify whether our framing may have yielded relatively low weights and how the methods used here can be simplified to be suitable for use in general public samples.

## 6 CONCLUSION

With the increasing importance of economic evaluations in health care, the question of how to best estimate health states valuations has become a crucial one. Conventional methodologies, such as TTO and SG, systematically arrived at different valuations of the same health state. PT may offer an explanation for this phenomenon (Bleichrodt, 2002), which was never tested directly. Using the nonparametric method (Abdellaoui et al., 2016), we demonstrated that it may be possible to significantly reduce these biases in HSVs. After correction for loss aversion, probability weighting, and utility curvature, TTO and SG weights for three health states were no longer different. This is an encouraging finding, but at the same time, the resulting low absolute values highlight the need for future research. Notwithstanding these important limitations, our findings do suggest the feasibility and relevance of this approach and may prove to be a first step in the move towards QALYs without bias.

## FUNDING SOURCE

This research did not receive any specific grant from funding agencies in the public, commercial, or not‐for‐profit sectors.

## CONFLICTS OF INTEREST

None.

## Supporting information

Data S1. Appendix S1: Overview of literature on correction for TTO and SG
Table A1. Overview of studies applying corrections to TTO and/or SG, with differences between methodologies and results categorized.
Appendix S2: Proofs for correction of TTO and SG
Appendix S3: Overview of experiment and counterbalancing procedures
Appendix S4: Elaborate formal description of measurement method
Appendix S5: Experimental instructions translated from Dutch and example screenshots.
Appendix S6: Experimental instructions translated from Dutch and example screenshots.
Online supplements: Isolated corrections with parametric assumptions
Table S1: Isolated effects of corrections for utility curvature (UC), loss aversion (LA) and probability weighting (PW) for TTO and SG weights [standard deviation in brackets].
