# Supplementary material for: QALYs without bias? Nonparametric correction of time trade‐off and standard gamble weights based on prospect theory
Source: Health Econ. 2019 Jun 24;28(7):843–54. doi: 10.1002/hec.3895 (PMC6618285; doi:10.1002/hec.3895)
Supplement: Supplementary file 1 — Data S1. Appendix S1: Overview of literature on correction for TTO and SG Table A1. Overview of studies applying corrections to TTO and/or SG, with differences between methodologies and results categorized. Appendix S2: Proofs for correction of TTO and SG Appendix S3: Overview of experiment and counterbalancing procedures Appendix S4: Elaborate formal description of measurement method Appendix S5: Experimental instructions translated from Dutch and example screenshots. Appendix S6: Experimental instructions translated from Dutch and example screenshots. Online supplements: Isolated corrections with parametric assumptions Table S1: Isolated effects of corrections for utility curvature (UC), loss aversion (LA) and probability weighting (PW) for TTO and SG weights [standard deviation in brackets]. [file HEC-28-843-s001.docx]

**Online supplementary file**

Lipman, Attema & Brouwer (2019) – QALYs without bias? Non-parametric correction of time trade-off and standard gamble weights based on prospect theory

**Contents:**

**Appendix A: Overview of literature on correction for TTO and SG**

**Appendix B: Proofs for correction of TTO and SG**

**Appendix C: Overview of experiment and counterbalancing procedures**

**Appendix D: Elaborate formal description of measurement method**

**Appendix E: Experimental instructions translated from Dutch and example screenshots**

**Appendix F: Isolated corrections with parametric assumptions**

**References**

**(Online) Appendix A: Overview of literature on correction for TTO and SG**

Table A1. Overview of studies applying corrections to TTO and/or SG, with differences between methodologies and results categorized.

| **Authors (by year)** | **Experiment** | **Measure corrected** | **Corrections** | **(Non-) Parametric** | **Effect** |
| --- | --- | --- | --- | --- | --- |
| Stiggelbout et al. ([1994](#_ENREF_18)) | N=31/D=2 | TTO | UC | PAR | TTO: + |
| Bleichrodt et al. ([1999](#_ENREF_12)) | N=172/D=1 | SG | PW | AS | SG: - |
| Martin et al. ([2000](#_ENREF_14)) | N=199/D=3 | TTO | UC | PAR | TTO: - |
| Bleichrodt et al. ([2001](#_ENREF_11)) | N=51/D=2 | SG | LA/PW | AS | SG: - |
| Bleichrodt ([2001](#_ENREF_9)) | N=66/D=4 | SG | PW | AS | SG - |
| Oliver ([2003](#_ENREF_15)) | N=30/D=3 | SG | LA/PW | AS | SG: - |
| Van Osch et al. ([2004](#_ENREF_21)) | N=45/D=6 | both | *SG*: LA/PW  *TTO:* UC | SG: AS  TTO: PAR | SG: -  TTO: = |
| Van der Pol & Roux ([2005](#_ENREF_20)) | N= 111/D= 1 | TTO | UC | AS/PAR | TTO: + |
| Bleichrodt et al. ([2007](#_ENREF_10)) | N=65/D=2 | SG | LA/PW | AS | SG: - |
| Attema & Brouwer ([2008](#_ENREF_2)) | N=70/D=2 | TTO | UC | NPAR | TTO: + |
| Attema & Brouwer ([2009](#_ENREF_3)) | N=70/D=2 | TTO | UC | NPAR | TTO: + |
| Abellán-Perpiñán et al. ([2009](#_ENREF_16)) | N=80^†^ /D=2 | TTO | UC | PAR | TTO: - |
| Attema & Brouwer ([2010](#_ENREF_4)) | N=70/D=1 | TTO | UC | NPAR | TTO: + |
| Doctor et al. ([2010](#_ENREF_13)) | Meta-analysis | SG | LA/PW | AS | SG: - |
| Attema & Brouwer ([2012a](#_ENREF_5)) | N=83/D=1 | TTO | UC | NPAR | TTO: + |
| Attema & Brouwer ([2012b](#_ENREF_6)) | N=83/D=1 | TTO | UC | NPAR | TTO: + |
| Attema et al. ([2013](#_ENREF_8)) | N=159/D=6 | TTO | UC | NPAR | TTO: = |
| Pinto-Prades & Abellán-Perpiñán, ([2012](#_ENREF_17)) | N=65/D=2 | SG | LA/PW | AS^‡^ ^§^ | SG: - |
| Attema & Brouwer ([2014](#_ENREF_7)) | N=80-112 /D=5 | TTO | UC | NPAR | TTO: + |

**Note:** *N =* sample size*, D =* amount of health states, and the following abbreviations are used: LA (loss aversion), PW (probability weighting), UC (utility curvature or time preference), AS (assumptions about parameter estimates), PAR (parametric elicitation), NPAR (non-parametric elicitation), + (upward effect of correction, and – (downward effect of correction).

^†^ = between-subjects tariff estimation with total N=720 and total D=18, ^‡^ = parameters were obtained through median optimization. ^§^= based on the same data as Bleichrodt et al. (2007).

**(Online) Appendix B: Proofs for correction of TTO and SG**

Below we will provide proofs for Eq. 3 and Eq. 4. We will first show how TTO and SG indifferences are derived under the general QALY model, and show how without loss of generality we can rewrite these derivations under the general QALY model. Next we apply our extension of this model as described in Section 2, to obtain proofs for Eq. 3 and Eq. 4. Comparing proofs for rewritten derivations under the general QALY model and the derivation of Eq. 3 and Eq. 4 show how the general QALY model is a special case of our extended model based on PT is

***B1. TTO and SG weights in the general QALY model***

TTO typically involves indifferences of the form: $\left( \beta_{x},T_{x} \right)\sim\left( {FH,T}_{y} \right).$ If we apply the general QALY model this indifference is evaluated by:

$U\left( \beta_{x} \right)L\left( T_{x} \right)=U\left( FH \right)L\left( T_{y} \right),$ (B1)

with $U\left( FH \right)=1$, this allows derivation of TTO weights (i.e. $U\left( \beta_{x} \right))$, by:

$U\left( \beta_{x} \right)=L\left( T_{y} \right)/L\left( T_{x} \right).$ (B2)

SG typically involves indifferences of the form:$\left( \beta_{x},T_{x} \right)\sim\left( {FH,T}_{x} \right)_{p}(D).$If we apply the general QALY model (assuming EU), this indifference is evaluated by:

$U\left( \beta_{x} \right)L\left( T_{x} \right)=pU\left( FH \right)L\left( T_{x} \right)+\left( 1-p \right)D,$ (B3)

with $U\left( FH \right)=1$ and $U\left( D \right)=0$. This allows derivation of SG weights (i.e. $U\left( \beta_{x} \right))$, by:

$U\left( \beta_{x} \right)=p$. (B4)

***B2. Proof for Equation 3 – TTO***

The first extension of the general QALY model we apply is reference-dependence, which is the most fundamental break-away from the general QALY model. To derive Eq. 3, we assume that the time spent in reduced health status is the RP, i.e.$\left( \beta_{r}, T_{r} \right)=\left( \beta_{x},T_{x} \right)$ in scenario A. Compared to this RP, the time spent in full health yields improved health status but a loss in life duration. Hence, the indifference in scenario A, $\left( \beta_{x},T_{x} \right)\sim\left( {FH,T}_{y} \right)$, implies indifference between staying at the RP and an improvement in health status experienced for ${(T}_{y})$ years and a loss in life duration$\left( T_{y}-T_{x} \right).$Importantly, duration is determined relative to the RP ($T_{r}).$Hence, after incorporating reference-dependence the indifference $\left( \beta_{x},T_{x} \right)\sim\left( {FH,T}_{y} \right)$ is evaluated by:

$U\left( \beta_{x} \right)L\left( T_{x}-T_{r} \right)=\left( U\left( FH \right)-U\left( \beta_{x} \right) \right)\left( L\left( T_{y}-T_{r} \right)-L\left( T_{a}-T_{r} \right) \right)+U\left( \beta_{x} \right)\left( L\left( T_{y}-T_{r} \right)-L\left( T_{x}-T_{r} \right) \right)$. (B5)

Next, we apply our notational convenience of writing $T_{x}^{*}=T_{x}-T_{r}$, for any $T$. Furthermore, we incorporate sign-dependence (i.e. $L^{i}(T))$ as described in Section 2, Because compared to $T_{x}^{*}$, durations $T_{y}^{*}$and$T_{a}^{*}$ are losses, this yields:

$U\left( \beta_{x} \right)L^{+}\left( T_{x}^{*} \right)=\left( U\left( FH \right)-U\left( \beta_{x} \right) \right)\left( L^{-}\left( T_{y}^{*} \right)-L^{-}\left( T_{a}^{*} \right) \right)+U\left( \beta_{x} \right)\left( L^{-}\left( T_{y}^{*} \right)-L^{+}\left( T_{x}^{*} \right) \right)$, (B6)

Next, we multiply the loss in life duration $\left( T_{y}-T_{x} \right)$ with the loss aversion index to obtain:

$U\left( \beta_{x} \right)L^{+}\left( T_{x}^{*} \right)=\left( U\left( FH \right)-U\left( \beta_{x} \right) \right)\left( L^{-}\left( T_{y}^{*} \right)-L^{-}\left( T_{a}^{*} \right) \right)+U\left( \beta_{x} \right)\lambda\left( L^{-}\left( T_{y}^{*} \right)-L^{+}\left( T_{x}^{*} \right) \right).$ (B7)

We normalize throughout, as is common in applications of PT, such that the utility of staying at the RP is zero, i.e. $L^{+}\left( T_{x}^{*} \right)=0$, and the utility of full health is one, $U\left( FH \right)=1$, which yields:

$\left( 1-U\left( \beta_{x} \right) \right)\left( L^{-}\left( T_{y}^{*} \right)-L^{-}\left( T_{a}^{*} \right) \right)=-\lambda U\left( \beta_{x} \right)L^{-}\left( T_{y}^{*} \right).$ (B8)

Rearranging Eq. B8 gives:

$\frac{1-U\left( \beta_{x} \right)}{U\left( \beta_{x} \right)}$ = $\frac{-\lambda L^{-}\left( T_{y}^{*} \right)}{L^{-}\left( T_{y}^{*} \right)-L^{-}\left( T_{a}^{*} \right)}$. (B9)

Solving for $U\left( \beta_{x} \right)$ gives:

$U\left( \beta_{x} \right)$ =$\frac{L^{-}\left( T_{y}^{*} \right)-L^{-}\left( T_{a}^{*} \right)}{\left( 1-\lambda\right)L^{-}\left( T_{y}^{*} \right)-L^{-}\left( T_{a}^{*} \right)},$ (B10)

which gives Eq. 3 if we set $L^{-}\left( T_{a}^{*} \right)=-1$.

Importantly, under the general QALY model we can derive the same derivation for $U\left( \beta_{x} \right)$ as follows: we take Eq. B1, subtract $U(\beta_{x})L\left( T_{x} \right)$ from both sides to obtain $0=U\left( FH \right)L(T_{y})$-$U(\beta_{x})L\left( T_{x} \right).$Adding $U(\beta_{x})L\left( T_{y} \right)$ to both sides and rearranging yields $0=\left( U\left( FH \right)-U\left( \beta_{x} \right) \right)L(T_{y})$+$U\left( \beta_{x} \right)\left( L\left( T_{y} \right)-L\left( T_{x} \right) \right)$. Realizing that $T_{x}^{*}=T_{x}-T_{r}$, that $L\left( T_{y} \right)=L\left( T_{y}^{*} \right)-L\left( T_{a}^{*} \right)$ and that $L\left( T_{x} \right)=L\left( T_{x}^{*} \right)-L\left( T_{a}^{*} \right),$ gives Eq. B8 with $\lambda=1$ and $L\left( T \right)=L^{+}(T)=L^{-}(T)$. Simple rearranging will give Eq. B10 $,$ which shows that the general QALY model is a special case of the reference-dependent evaluation.

***B3. Proof for Equation 4 - SG***

We assume $\left( \beta_{x},T_{x} \right)$ is the reference point (i.e. $\left( \beta_{r}, T_{r} \right)=\left( \beta_{x},T_{x} \right))$. If we introduce reference-dependence, the gamble $\left( {FH,T}_{x} \right)_{p}(D)$ then consists of the option to gain quality of life for $T_{x}-T_{a}$ years with a probability *p*, and a risk of death after $T_{a}$years with probability $1-p$, which is a loss in lifetime of $T_{a}-T_{x}$ years in health state$\beta_{x}$. Again, these durations are determined relative to $T_{r}$.Thus, when the respondent is indifferent between the two options, the following equation holds:

$U\left( \beta_{x} \right)L\left( T_{x}-T_{r} \right)=p\left( U(FH)-U(\beta_{x}) \right)\left( L\left( T_{x}-T_{r} \right)- L\left( T_{a}-T_{r} \right) \right)$ + $\left( 1-p \right)U\left( \beta_{x} \right)(L\left( T_{a}-T_{r} \right)-L\left( T_{x}-T_{r} \right))$**.**  (B11)

Second, we introduce probability weighting, by assigning sign-dependent decision weights (i.e. $w^{i}(p)$) to probabilities, i.e.:

$U\left( \beta_{x} \right)L\left( T_{x}-T_{r} \right)=w^{+}(p)\left( U(FH)-U(\beta_{x}) \right)\left( L\left( T_{x}-T_{r} \right)- L\left( T_{a}-T_{r} \right) \right)$ + $w^{-}\left( 1-p \right)U\left( \beta_{x} \right)(L\left( T_{a}-T_{r} \right)-L\left( T_{x}-T_{r} \right))$**.**

Next, we incorporate sign-dependence for $L^{i}\left( T \right)$ as described in Section 2, and apply our notational convenience of writing $T_{x}^{*}=T_{x}-T_{r}$, for any $T$. Because compared to $T_{x}^{*}$, duration $T_{a}^{*}$ is a loss, this yields:

$U\left( \beta_{x} \right)L^{+}\left( T_{x}^{*} \right)=w^{+}(p)\left( U(FH)-U(\beta_{x}) \right)\left( L^{+}\left( T_{x}^{*} \right)- L^{-}\left( T_{a}^{*} \right) \right)$ + $w^{-}\left( 1-p \right)U\left( \beta_{x} \right)\left( L^{-}\left( T_{a}^{*} \right)-L^{+}\left( T_{x}^{*} \right) \right).$ (B12)

Next, we multiply the loss in life duration (i.e. immediate death) with loss aversion to obtain:

$$U\left( \beta_{x} \right)L^{+}\left( T_{x}^{*} \right)=w^{+}\left( p \right)\left( U\left( FH \right)-U\left( \beta_{x} \right) \right)\left( L^{+}\left( T_{x}^{*} \right)- L^{-}\left( T_{a}^{*} \right) \right)+$$

$w^{-}\left( 1-p \right) U\left( \beta_{x} \right)\lambda(L^{-}\left( T_{a}^{*} \right)-L^{+}\left( T_{x}^{*} \right)).$ (B13)

We assume throughout, as is common in applications of PT, that the utility of staying at the RP, i.e. $L^{+}\left( T_{x}^{*} \right)=0$, and$U\left( FH \right)=1$ which yields, after rearranging:

$\left( 1-U\left( \beta_{x} \right) \right)w^{+}(p)\left( -L^{-}\left( T_{a}^{*} \right) \right)=-\lambda U\left( \beta_{x} \right)w^{-}(1-p)(L^{-}\left( T_{a}^{*} \right)).$ (B14)

Rearranging Eq. B14 gives:

$\frac{1-U\left( \beta_{x} \right)}{U\left( \beta_{x} \right)}$ = $\frac{-\lambda w^{-}\left( 1-p \right)L^{-}\left( T_{a}^{*} \right)}{w^{+}(p)\left( -L^{-}\left( T_{a}^{*} \right) \right)}$ (B15)

Solving for $U\left( \beta_{x} \right)$ gives:

$U\left( \beta_{x} \right)$ =$\frac{w^{+}(p)\left( -L^{-}\left( T_{a}^{*} \right) \right)}{-\lambda w^{-}\left( 1-p \right)L^{-}\left( T_{a}^{*} \right)+w^{+}(p)\left( -L^{-}\left( T_{a}^{*} \right) \right)},$ (B16),

which gives Eq. 4 if we set $L^{-}\left( T_{a}^{*} \right)=-1$.

Again, we can obtain a similar derivation for $U\left( \beta_{x} \right)$ under the general QALY: we take Eq. B3A, subtract $U(\beta_{x})L\left( T_{x} \right)$ from both sides to obtain $0=pU\left( FH \right)L\left( T_{x} \right)$-$U(\beta_{x})L\left( T_{x} \right).$Adding $pU(\beta_{x})L\left( T_{x} \right)$ to both sides and rearranging yields $0=p\left( 1-U\left( \beta_{x} \right) \right)L(T_{x})-(1-p)U\left( \beta_{x} \right)\left( L\left( T_{x} \right) \right)$. Realizing that $T_{x}={-T}_{a}^{*}$, gives Eq. B14 with $\lambda=1$ and $w^{+}\left( p \right)=w^{-}(p)=p$. Rearranging then gives Eq. B16, which shows how the general QALY model is a special case of the reference-dependent evaluation.

**B3. On the scaling of** $\boldsymbol{L}^{\boldsymbol{-}}\left( \boldsymbol{T}_{\boldsymbol{a}}^{\boldsymbol{*}} \right)$

We have shown how Eq. 3 and Eq. 4 can be derived from Eq. B8 and B16 respectively, scaling such that $L^{-}\left( T_{a}^{*} \right)=-1.$ Given that $L^{i}\left( T^{*} \right)$ is cardinal, we are allowed to freely set the utility of any two points along this scale. We already, as in conventional in applications of PT, set the utility of the RP to 0, i.e. $L^{+}\left( T_{x}^{*} \right)=0$, and, hence, we are allowed to specify the utility of 1 other point. Given that $T_{a}$ is the lowest possible outcome, we believe it is natural to assign this outcome $L^{-}\left( T_{a}^{*} \right)=-1$. This scaling yields Eq. 3 and Eq. 4 but it is straightforward to show that this scaling is immaterial to the derivation of TTO and SG weights (it matters only for utility elicitation). Take, for example, a subject with $\lambda=2$, $p=0.8,$ $w^{+}\left( 0.8 \right)=0.9, w^{-}\left( 1-0.8 \right)=0.25,$and $L^{-}\left( T_{y}^{*} \right)=-0.5.$ Multiplication of $L^{i}\left( T^{*} \right)$by any non-zero constant does not affect TTO or SG weights, and any addition or subtraction satisfying$L^{-}\left( T_{a}^{*} \right)\neq0$, leaves TTO or SG weights unaffected.

**(Online) Appendix C: Overview of experiment and counterbalancing procedures**

First, the order of the two parts of the experiment (*health state valuation vs. non-parametric method*) was counterbalanced. Within the utility elicitation part, the order in which participants faced gains or losses was randomized; half of the participants always completed gain sections first, whilst the other half completed loss sections first. Furthermore, within the health state valuation part, the order in which TTO and SG were presented was randomized, as was the order of the health states. Furthermore, a total of five practice blocks and four consistency checks were used.

| **Part 1: Health state valuation** |  | **Part 2: Non-parametric method** |
| --- | --- | --- |
| Practice TTO ($\beta_{p}$) |  | *Practice block 1:* Certainty equivalents |
| TTO ($\beta_{1}$, $\beta_{2}$, $\beta_{3}$) |  | *Stage 1*: connecting $L^{+}\left( T^{*} \right)$ *and* $L^{-}\left( T^{*} \right)$ |
| Practice SG ($\beta_{p}$) |  | *Practice block 2:* Trade-off method |
| SG ($\beta_{1}$, $\beta_{2}$, $\beta_{3}$) |  | *Stage 2*: elicitation of $L^{+}\left( T^{*} \right)$ |
|  |  | *Consistency block*: Re-elicitation of $x_{2}^{+}$ |
|  |  | *Stage 3:* elicitation of $L^{-}\left( T^{*} \right)$ |
|  |  | *Consistency block*: Re-elicitation of $x_{2}^{-}$ |
|  |  | *Stage 4a:* probability weighting gains $w^{+}\left( p \right)$ |
|  |  | *Consistency block*: Re-elicitation of randomly selected $x_{p}^{+}$ |
|  |  | *Stage 4b:* probability weighting losses $w^{+}\left( p \right)$ |
|  |  | *Consistency block*: Re-elicitation of randomly selected $x_{p}^{+}$ |

**(Online) Appendix D: Elaborate formal description of measurement method**

Abdellaoui and colleagues ([2016](#_ENREF_1)) describe the following three-stage methodological procedure, here appended to reflect decision under risk (see Table D1 for stimuli used in this adaptation of their method). Unless otherwise specified, the assumptions presented in Section 2 of this paper will hold. Importantly, we will simplify notation in this Appendix, to ensure comparability to the original authors’ work ([Abdellaoui et al., 2016](#_ENREF_1)). All outcomes refer to health profiles of the form $\left( \beta,T \right)$, which are evaluated as in Eq. 1 and Eq. 2. Prospects are rank-ordered for duration, which indicates that for gain outcomes $\left( \beta_{x}, T_{x}^{*} \right)_{p}(\beta_{y}, T_{y}^{*})$ signifies that $T_{x}\geq T_{y}$ and for loss outcomes it signifies that $T_{x}^{*}$ $<T_{y}^{*}.$For prospects containing gain and loss outcomes (i.e. mixed prospects), $\left( \beta_{x},T_{x}^{*} \right)_{p}(\beta_{y}, T_{y}^{*})$ signifies that$T_{x}^{*}$ is a gain and $T_{y}^{*}$is a loss. Throughout the whole application of this method, $\beta_{x}$ will remain constant (i.e. at full health), and will thus cancel out in the evaluation of each indifference mentioned in this Appendix. For example the indifference, $\left( \beta_{x}, T_{x}^{*} \right)_{p}\left( \beta_{x}, T_{y}^{*} \right)\sim\left( \beta_{x},T_{z}^{*} \right)$, with $T_{y}^{*}<0<T_{z}^{*}< T_{x}^{*}$, will be evaluated as:

$w^{+}\left( p \right){U\left( \beta_{x} \right)L}^{+}\left( T_{x}^{*} \right)+w^{-}\left( 1-p \right)U\left( \beta_{x} \right)L^{-}\left( T_{y}^{*} \right)={U\left( \beta_{x} \right)L}^{+}\left( T_{z}^{*} \right)$,

where dividing by $U\left( \beta_{x} \right)$ gives:

$w^{+}\left( p \right)L^{+}\left( T_{x}^{*} \right)+w^{-}\left( 1-p \right)L^{-}\left( T_{y}^{*} \right)=L^{+}\left( T_{z}^{*} \right)$.

For brevity, we will suppress $U\left( \beta_{x} \right)$ in the derivations of the non-parametric method from here onwards (i.e. D1-D8), and instead apply the general notation also used by Abdellaoui and colleagues ([2016](#_ENREF_1)). Hence, all outcomes are health profiles, i.e.:$x_{0}$(reference point),$l,\mathcal{l,}\mathcal{L}, g,\mathcal{g, G}$, ${[x}_{1}^{+},x_{2}^{+},\ldots,x_{k_{G}}^{+}]$and $[x_{1}^{-},x_{2}^{-},\ldots,x_{k_{L}}^{-}]$ refer to $T^{*}$life years in $\beta_{x}$.

D.1. First stage: connecting $L^{+}\left( T^{*} \right)$ *and* $L^{-}\left( T^{*} \right)$

First, we select a probability *p* that is kept constant throughout the first three stages and a gain $g$. Next, $l$ is elicited, by means of the following indifference$g_{p}l\sim x_{0}$.

Equation (1) thus implies that:

$w^{+}\left( p \right)L^{+}\left( g \right)+w^{-}\left( 1-p \right)L^{-}\left( l \right)=L^{+}\left( x_{0} \right)=0$.  **(D1)**

Then, the certainty equivalents $x_{1}^{+}$ and $x_{1}^{-}$ are elicited, by the following indifferences: $x_{1}^{+}\sim g_{p}x_{0}$ and $x_{1}^{-}\sim l_{p}x_{0}$. These indifferences consequently imply:

$L^{+}\left( x_{1}^{+} \right)=w^{+}\left( p \right)L^{+}\left( g \right),$ **(D2)**

and equivalently for losses:

$L^{-}\left( x_{1}^{-} \right)=w^{-}\left( p \right)L^{-}\left( l \right)$. **(D3)**

Combining Eqs. (D1) − (D4) gives:

$L^{+}\left( x_{1}^{+} \right)=-L^{-}\left( x_{1}^{-} \right)$. **(D4)**

Through this equation we obtain the first elements of the standard sequence ($x_{1}^{+}$ and $x_{1}^{-}$ ), which is elicited in subsequent stages.

D.2. Second and third stage: elicitation of $L^{+}\left( T^{*} \right)$ *and* $L^{-}\left( T^{*} \right).$

Next, the trade-off method by [Wakker and Deneffe (1996](#_ENREF_22)) is employed to elicit a standard sequence. Let $\mathcal{l}$ be a prespecified loss. First, subjects are presented with the prospects ${x_{1}^{+}}_{p}\mathcal{L}$ and $\mathcal{l}_{p}x_{0}$, in order to elicit the loss $\mathcal{L}$ for which subjects are indifferent ($x_{1}^{+}$ is the gain from stage 1). The indifference ${x_{1}^{+}}_{p}\mathcal{L\sim}{\mathcal{l}_{p}x}_{0}$ gives:

$w^{+}\left( p \right)L^{+}\left( x_{1}^{+} \right)+w^{-}\left( p \right)L^{-}\left( \mathcal{L} \right)=w^{-}\left( p \right)L^{-}\left( \mathcal{l} \right)$. **(D5)**

Through rearranging Eq. (D5) we obtain,

$L^{+}\left( x_{1}^{+} \right)-L^{+}\left( x_{0} \right)=\frac{w^{-}\left( p \right)}{w^{+}\left( p \right)}\left( L^{-}\left( \mathcal{l} \right)-L^{-}\left( \mathcal{L} \right) \right)$. **(D6)**

Second, subjects are presented with the prospects ${x_{2}^{+}}_{p}\mathcal{L}$and ${x_{1}^{+}}_{p}\mathcal{l}$, where the gain $x_{2}^{+}$ is varied such that they are indifferent ${x_{2}^{+}}_{p}\mathcal{L\sim}{x_{1}^{+}}_{p}\mathcal{l}$. This indifference implies, after rearranging:

$L^{+}\left( x_{2}^{+} \right)-L^{+}\left( x_{1}^{+} \right)=\frac{w^{-}\left( p \right)}{w^{+}\left( p \right)}\left( L^{-}\left( \mathcal{l} \right)-L^{-}\left( \mathcal{L} \right) \right)$. **(D7)**

Combining Eqs. (D6) and (D7) gives:

$L^{+}\left( x_{2}^{+} \right)-L^{+}(x_{1}^{+})=L^{+}\left( x_{1}^{+} \right)-L^{+}(x_{0})$. **(D8)**

Finally, we elicit a series of indifferences: ${x_{j}^{+}}_{p}\mathcal{L\sim}{x_{j-1}^{+}}_{p}\mathcal{l,}j=2,\ldots,k_{G}$, which together form the standard sequence ${[x_{0,} x}_{1}^{+},x_{2}^{+},\ldots,x_{k_{G}}^{+}]$ for gains. It follows straightforwardly that for all $j$, $L^{+}\left( x_{j}^{+} \right)-L^{+}(x_{j-1}^{+})=L^{+}\left( x_{1}^{+} \right)-L^{+}(x_{0})$.

The standard sequence for losses is constructed equivalently (part three). Subjects face similar prospects, where we first fix a gain $\mathcal{g}$ to elicit the gain $\mathcal{G}$ such that subjects produce the following indifference: $\mathcal{G}_{p}x_{1}^{-}\sim\mathcal{g}_{p}x_{0}$. Finally, we elicit the standard sequence

$[x_{0,}x_{1}^{-},x_{2}^{-},\ldots,x_{k_{L}}^{-}]$ as described above using the following general form: $\mathcal{G}_{p}x_{j}^{-}\sim\mathcal{g}_{p}x_{j-1}^{-}$, $j=2,\ldots,k_{L}.$

D.3. Fourth stage: probability weights

To measure the probability weighting functions $w^{+}(p)$ and $w^{-}(p)$, we asked for the certainty equivalents $x_{p}^{+}$ and $x_{p}^{-}$ of the prospects $x_{{kG}_{p}}^{+}x_{0}$ and $x_{{kL}_{p}}^{-}x_{0}$. The outcomes $x_{k_{G}}^{+}$ and $x_{k_{G}}^{-}$ are the maximum (minimum) outcome elicited in the standard sequence. Therefore, it follows from the probability weighting function and the chosen scaling of utility that $L^{+}\left( x_{P}^{+} \right)=w^{+}(p)$ and $-L^{-}\left( x_{p}^{-} \right)=w^{-}(p)$. The values of $L^{+}\left( x_{p}^{+} \right)$ and $L^{-}\left( x_{p}^{-} \right)$ are interpolated from their respective standard sequences (elicited in stage 2 and 3). The probability $p$ was varied (0.1; 0.3; 0.5; 0.7; 0.9) to measure the probability weighting functions for a wide range of probabilities, both for gains and losses.

**Table D1: Four-stage procedure to measure utility of life duration**

The third column shows the variables assessed in each stage, and column four shows the elicited indifferences. The fifth column shows the implication of these elicited indifferences. The final column shows the stimuli used in this experiment.

|  | Elicited | Indifference | Implication | Stimuli |
| --- | --- | --- | --- | --- |
| Stage 1 | $l$ | $g_{p}l\sim x_{0}$ | $L^{+}\left( x_{1}^{+} \right)=-L^{-}\left( x_{1}^{-} \right)$ | $g=5 years$  $p=$ 0.5  $x_{0}=70 years$ |
|  | $x_{1}^{+}$ | $x_{1}^{+}\sim g_{p}x_{0}$ |  |  |
|  | $x_{1}^{-}$ | $x_{1}^{-}\sim l_{p}x_{0}$ |  |  |
| Stage 2 | $\mathcal{L}$ | ${x_{1}^{+}}_{p}\mathcal{L\sim}\mathcal{l}_{p}x_{0}$ | $L^{+}\left( x_{j}^{+} \right)-L^{+}(x_{j-1}^{+})=L^{+}\left( x_{1}^{+} \right)-L^{+}(x_{0})$ | $\mathcal{l=}-1 year$ |
|  | $x_{j}^{+}$ | ${x_{j}^{+}}_{p}\mathcal{L\sim}{x_{j-1}^{+}}_{p}\mathcal{l}$ |  |  |
| Stage 3 | $\mathcal{G}$ | $\mathcal{G}_{p}x_{1}^{-}\sim\mathcal{g}_{p}x_{0}$ | $L^{-}\left( x_{j}^{-} \right)-L^{-}\left( x_{j-1}^{-} \right)=L^{-}\left( x_{1}^{-} \right)-L^{+}\left( x_{0} \right)$ | $\mathcal{g=}1 year$ |
|  | $x_{j}^{-}$ | $\mathcal{G}_{p}x_{j}^{-}\sim\mathcal{g}_{p}x_{j-1}^{-}$ |  |  |
| Stage 4 | $x_{p}^{+}$ | $x_{p}^{+}\sim{x_{kG}^{+}}_{p}x_{0}$ | $L^{+}\left( x_{p}^{+} \right)/L^{+}\left( x_{kG}^{+} \right)=w^{+}(p)$ | $p =\left\{ \begin{matrix} \begin{matrix} 0.1 \\ 0.3 \\ 0.5 \end{matrix} \\ 0.7 \\ 0.9 \end{matrix} \right.$ |
|  | $x_{p}^{-}$ | $x_{P}^{-}\sim x_{{kL}_{p}}^{-}x_{0}$ | $L^{-}\left( x_{p}^{-} \right)/L^{-}\left( x_{kL}^{-} \right)=w^{-}(p)$ | $p=\left\{ \begin{matrix} \begin{matrix} 0.1 \\ 0.3 \\ 0.5 \end{matrix} \\ 0.7 \\ 0.9 \end{matrix} \right.$ |

**(online) Appendix E: Experimental instructions translated from Dutch and example screenshots.**

**E1. Utility elicitation**

The following instruction was used to introduce the non-parametric method:

‘Imagine you will live until 70 years old in perfect health. After becoming 70 you will contract a deadly disease, which will lead to a direct, painless death.

As a result of recent developments in pharmacological sciences, several drugs have become available. Your task involves comparing these drugs, and indicating your preferred drug. In any case you will be comparing two drugs. The drugs are described as Drug A and Drug B. In some cases you will have the opportunity to take a drug that has no effect on your health, as if you would be taking a placebo pill.

For some drugs it is not yet entirely clear if they work, picking these drugs involves some risk. For example, Drug B has 50% chance of increasing your length of life by 4 years, with no adverse effects or other consequences if the Drug fails. It cannot be determined beforehand for whom the drugs will work, you can expect this is determined solely by chance. Personal characteristics have no effect on this chance, so your chances are just as good as any others’.

For some drugs, it is known that if they are ineffective, they could have a negative influence on your health. Using these types of drugs could potentially lead to a reduction in length of life. The result of taking these drugs will be determined immediately by a physician, but its’ results are final. No new or better drugs will be developed to change your situation.

After you compared Drug A and B with each other, you will be asked to indicate by means of a slider when you would be indifferent between Drug A and B.’

*E1.1. Stages of non-parametric method*

Considering the non-parametric method is chained, i.e. answers from the previous stage carry over to the next meaning that differences could exist between subjects. In Table D1, example instructions can be found with some screenshots presenting the visual representation for each stage, where outcomes were likely to be different for each individual subject. Appendix D lists how these outcomes were obtained from revealed preferences.

**Table E1**: Example instructions per stage of the non-parametric method, with indifference elicited (for implications, see Appendix D)

| *Stage 1: connecting* $L^{+}\left( T^{*} \right)$ *and* $L^{-}\left( T^{*} \right)$ *–* Mixed prospect - $g_{p}l\sim x_{0}$ |
| --- |
| ‘You have the choice between a remedy (Drug A) that can both increase and decrease your length of life, and a remedy (Drug B) that does not change your situation. If you pick Drug A, you have a chance of 50% to gain 5 life years. If the drug is ineffective, you lose some life years. If you pick Drug B, your situation remains unchanged and you will live until 70. |
| *Stage 1: connecting* $L^{+}\left( T^{*} \right)$ *and* $L^{-}\left( T^{*} \right)$ *–* CE gains - $x_{1}^{+}\sim g_{p}x_{0}$ |
| ‘Now you have to make a choice between two drugs that will not affect your quality of life, but only your length of life. Drug B will increase your length of life with a couple of years, while if you pick Drug A a 50% chance exists that your length of life will not be increased, with a 50% chance of increasing your length of life with 5 years. |
| *Stage 1: connecting* $L^{+}\left( T^{*} \right)$ *and* $L^{-}\left( T^{*} \right)$ *–* CE losses - $x_{1}^{-}\sim l_{p}x_{0}$ |
| Again, imagine you would live until 70 in perfect health, after which you would die immediately and painlessly, as described before. However, you have contracted another fatal disease that should also be treated. You have to choose between drugs that will not affect your quality of life, but will affect your length of life to some extent.  Two drugs exist, Drug A will reduce your length of life with some years, while if you pick Drug B, a 50% chance exists of reducing your lifetime by **3 years** and a 50% chance that your lifetime will not be reduced at all. |
| *Stage 2: Standard sequence gains* $L^{+}\left( T^{*} \right)$ *– Eliciting offset loss -* ${x_{1}^{+}}_{p}\mathcal{L\sim}\mathcal{l}_{p}x_{0}$ |
| In this next phase, you compare (as in the practice block) two drugs that both carry a degree of risk. Drug A has a 50% chance of retaining your expected health (70 years), and a 50% chance of you losing 1 year. Drug B has a chance of improving your life expectancy by some years, but may also reduce your lifetime by a few years. |
| *Stage 2: Standard sequence gains* $L^{+}\left( T^{*} \right)$ *– Standard sequence -* ${x_{j}^{+}}_{p}\mathcal{L\sim}{x_{j-1}^{+}}_{p}\mathcal{l}$ |
| Now, you will compare several series of drugs with each other. Both drugs may each time both increase your health to a different extent. Additionally, both drugs will have risks of adverse effects, and thus decrease your lifetime. In each series you are asked to pick your preferred drug and specify a value for which you would be indifferent. |
| *Stage 3: Standard sequence losses* $L^{-}\left( T^{*} \right)$ *– Eliciting offset gains -* $\mathcal{G}_{p}x_{1}^{-}\sim\mathcal{g}_{p}x_{0}$ |
| Imagine again that you would become 70 in perfect health, followed by immediate and painless death as a result form the disease described earlier. Yet, you have contracted another disease that is also to be treated. You have to choose between Drugs that do not affect your quality of life, but may affect your length of life.  In this phase you compare two drugs for this second disease, which both carry some degree of risk. Drug A has a 50% chance of you retaining your current health (70 years), and a 50% chance of gaining 1 year. Drug B has the chance of improving your length of life, but may also reduce your life time by a few years. |
| *Stage 3: Standard sequence losses* $L^{-}\left( T^{*} \right)$ *– Standard sequence -* $\mathcal{G}_{p}x_{j}^{-}\sim\mathcal{g}_{p}x_{j-1}^{-}$ |
| Next, you will compare series of Drugs with each other. Both drugs may each time both decrease or increase your health. You will be asked which Drug you pick, after which you will be asked to select that value which would make you indifferent between both Drugs. |
| *Stage 4: Probability weighting for gains* $w^{+}\left( p \right)$ *-* $x_{p}^{+}\sim{x_{kG}^{+}}_{p}x_{0}$ |
| In this next part, you are again asked to compare different Drugs. Now, however, the drugs may have different chances of success. Your choice is between Drug A with a risky outcome, and Drug B which has a certain outcome. Eventually you will be asked to indicate when you would be indifferent between these Drugs. Please imagine that if your situation does not change, you would live until 70 years old, followed by immediate, painless death. |
| *Stage 4: Probability weighting for gains* $w^{-}\left( p \right)$ *-* $x_{p}^{-}\sim x_{{kL}_{p}}^{-}x_{0}$ |
| Imagine: You would live until 70 years old in perfect health, followed by immediate painless death, as described before. However, you’ve contracted another unrelated disease that needs to be treated. Your choice in between two Drugs that treat this second disease, which will not affect quality of life, but only your length of life to some extent. One of these has the chance of returning you to your initial expected health (age 70), with a varying chance of success. The other Drug will reduce your life duration with some years for certain. |


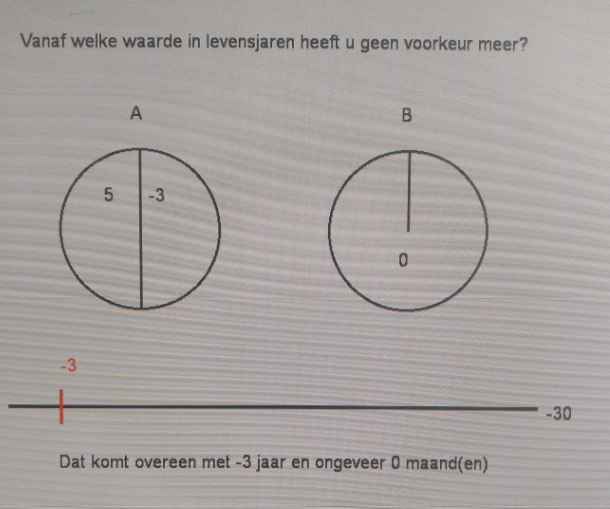


**Figure E1: Example screenshot First stage (slider screen)**


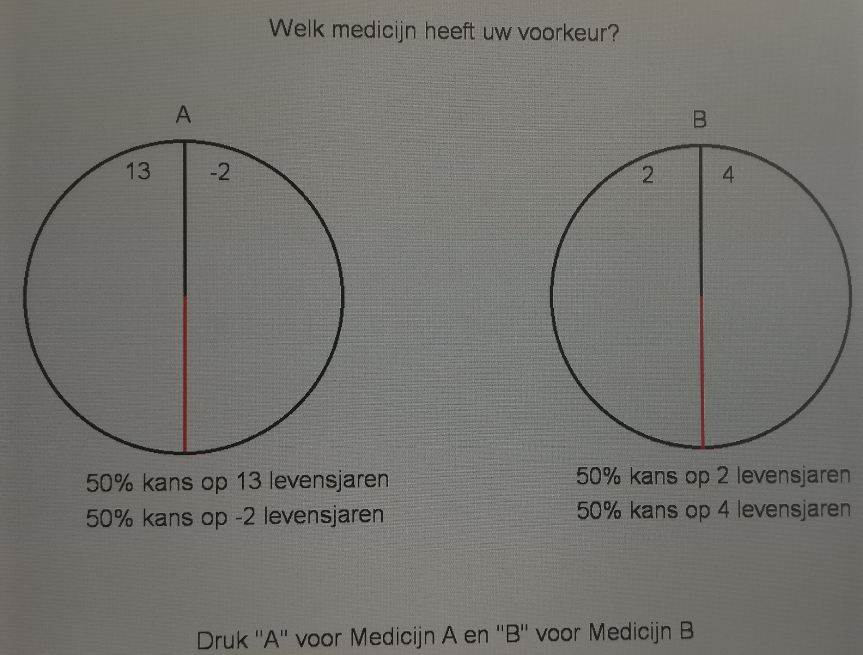


**Figure E2: Example screenshot second stage (choice Screen)**

**E2. Health state valuation**

The following instruction was used to introduce time trade-off (TTO) and standard gamble (SG):

‘In this next part you will compare several Treatments with each other, that will to some extent affect your quality of life. Throughout this part of the experiment, please imagine the following: You will live until age 50 in perfect health. After becoming 50, you will contract a disease, forcing you to choose between two different Treatments, which we will present to you.

You are tasked with comparing treatments and indicating your preferred Treatment. We will describe them as Treatment A and Treatment B. In some cases it is not yet known beforehand if treatments will be effective, and for whom. It cannot be determined beforehand for whom the Treatments will work, you can expect this is determined solely by chance. Personal characteristics have no effect on this chance, so your chances are just as good as any others’. The result of these Treatments will be determined immediately by a physician, but its’ results are final. No new or better Treatments will be developed to change your situation.

*E2.1. TTO instruction and screenshot*

In this part you compare two Treatments affecting your length of life and quality of life. If you pick Treatment Am you will live for some more years after age 50 in perfect health, followed by immediate, painless death. If you choose Treatment B, you will live 20 more years after age 50 in a reduced quality of life.


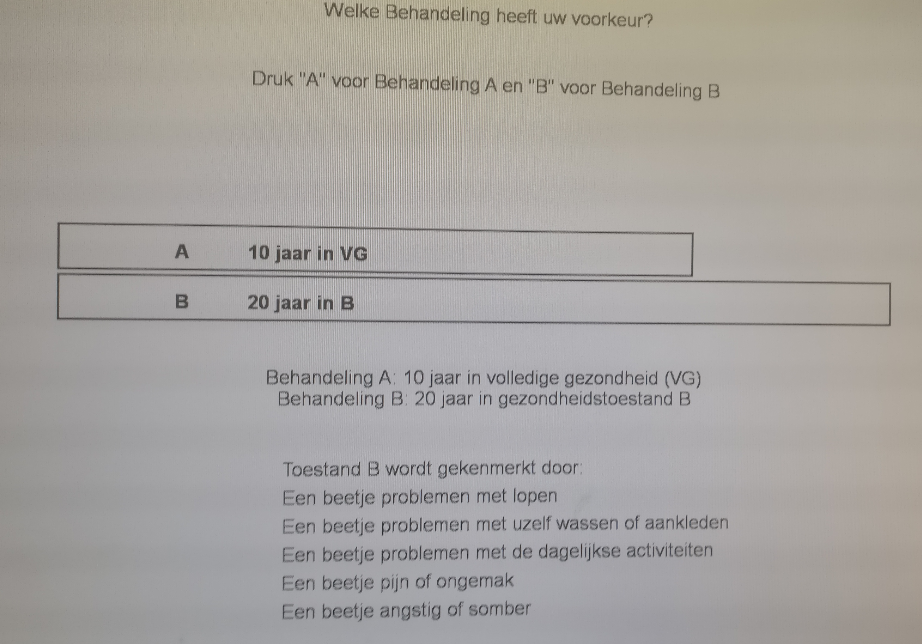


Figure E1: Screenshot of TTO choice screen

*E2.2 SG instruction and screenshot*

In this part you compare two Treatments of which 1 has a certain outcome, while the other has a risky outcome. If you pick the certain, you will live 20 more years after age 50 in a reduced quality of life. The risky treatment offers you the chance to live 20 more years after age 50 in perfect health. However, if this treatment is ineffective, you will die immediately and painlessly after age 50.


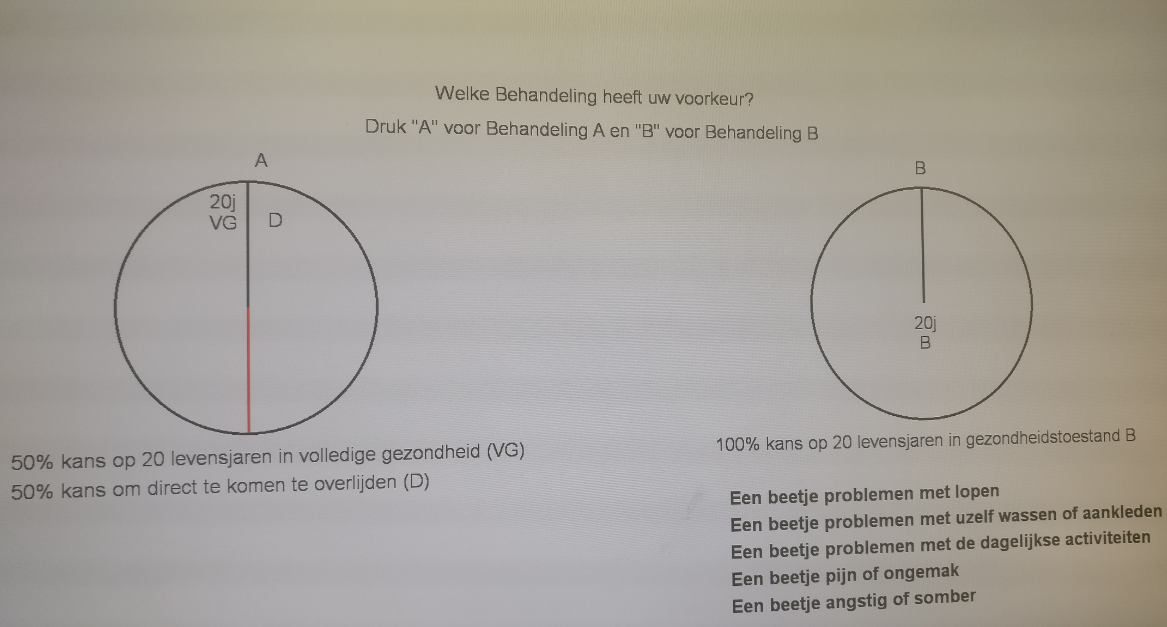


Figure E2: Screenshot of SG choice screen

**(online) Appendix F: Isolated corrections with parametric assumptions**

We also performed isolated corrections with parametric assumptions, i.e. where$L$ and $w$ were estimated by means of a power function and Tversky and Kahneman’s ([1992](#_ENREF_19)) one-parameter weighting function respectively. First, we corrected TTO for utility curvature only, with λ = 1. Second, TTO weights were corrected for loss aversion only, with linear utility (i.e. $L^{i}\left( T^{*} \right)=T^{*})$. Third, we corrected SG for probability weighting only, with λ = 1. Finally, SG weights were corrected for loss aversion only, with $w^{i}\left( p \right)=p$. This allow us to demonstrate the influence of each correction in isolation. Table S1 shows similar results compared to our findings for non-parametric corrections. For TTO, correcting for loss aversion had a stronger downward influence than correcting for curvature of$L^{i}\left( T^{*} \right)$. For SG, this finding also holds, i.e. correction for loss aversion had a stronger affect than correcting for probability weighting. Again, this confirms our conclusion that relative ineffectiveness of correction based on parametric estimation appears to be driven by the estimation of$w^{i}(p).$

***Table S1:* Isolated effects of corrections for utility curvature (UC), loss aversion (LA) and probability weighting (PW) for TTO and SG weights [standard deviation in brackets].**

| Health state | Uncorrected  weight |  | UC only |  | LA only |  | PW only |  |
| --- | --- | --- | --- | --- | --- | --- | --- | --- |
|  | | | | | | | | |
| TTO: Implication | $\lambda=1 \& L^{i}\left( T^{*} \right)=T^{*}$ | | $\lambda=1$ | | $L^{i}\left( T^{*} \right)=T^{*}$ | |  | |
| $\beta_{1}$ : 21211 | 0.665 | [0.268] | 0.611 | [0.296] | 0.537 | [0.311] |  |  |
| $\beta_{2} :$31221 | 0.605 | [0.259] | 0.558 | [0.287] | 0.474 | [0.3] |  |  |
| $\beta_{3} :$ 32341 | 0.39 | [0.259] | 0.365 | [0.277] | 0.288 | [0.259] |  |  |
|  |  |  |  |  |  |  |  |  |
| SG: Implication | $\lambda=1 \& w^{i}\left( p \right)=p$ | |  |  | $w^{i}\left( p \right)=p$ | | $\lambda=1$ | |
| $\beta_{1}$ : 21211 | 0.75 | [0.25] |  |  | 0.63 | [0.307] | 0.715 | [0.271] |
| $\beta_{2} :$31221 | 0.706 | [0.261] |  |  | 0.584 | [0.305] | 0.676 | [0.287] |
| $\beta_{3} :$ 32341 | 0.518 | [0.276] |  |  | 0.387 | [0.278] | 0.502 | [0.306] |

**References**

ABDELLAOUI, M., BLEICHRODT, H., L’HARIDON, O. & VAN DOLDER, D. 2016. Measuring Loss Aversion under Ambiguity: A Method to Make Prospect Theory Completely Observable. *Journal of Risk and Uncertainty,* 52**,** 1-20.

ATTEMA, A. E. & BROUWER, W. B. 2008. Can we fix it? Yes we can! But what? A new test of procedural invariance in TTO‐measurement. *Health Economics,* 17**,** 877-885.

ATTEMA, A. E. & BROUWER, W. B. 2009. The correction of TTO-scores for utility curvature using a risk-free utility elicitation method. *Journal of health economics,* 28**,** 234-243.

ATTEMA, A. E. & BROUWER, W. B. 2010. On the (not so) constant proportional trade-off in TTO. *Quality of Life Research,* 19**,** 489-497.

ATTEMA, A. E. & BROUWER, W. B. 2012a. Constantly proving the opposite? A test of CPTO using a broad time horizon and correcting for discounting. *Quality of Life Research,* 21**,** 25-34.

ATTEMA, A. E. & BROUWER, W. B. 2012b. The way that you do it? An elaborate test of procedural invariance of TTO, using a choice-based design. *The European Journal of Health Economics,* 13**,** 491-500.

ATTEMA, A. E. & BROUWER, W. B. 2014. Deriving time discounting correction factors for TTO tariffs. *Health economics,* 23**,** 410-425.

ATTEMA, A. E., VERSTEEGH, M. M., OPPE, M., BROUWER, W. B. & STOLK, E. A. 2013. Lead time TTO: leading to better health state valuations? *Health economics,* 22**,** 376-392.

BLEICHRODT, H. 2001. Probability weighting in choice under risk: an empirical test. *Journal of Risk and Uncertainty,* 23**,** 185-198.

BLEICHRODT, H., ABELLAN-PERPIÑAN, J. M., PINTO-PRADES, J. L. & MENDEZ-MARTINEZ, I. 2007. Resolving Inconsistencies in Utility Measurement Under Risk: Tests of Generalizations of Expected Utility. *Management Science,* 53**,** 469-482.

BLEICHRODT, H., PINTO, J. L. & WAKKER, P. P. 2001. Making descriptive use of prospect theory to improve the prescriptive use of expected utility. *Management science,* 47**,** 1498-1514.

BLEICHRODT, H., VAN RIJN, J. & JOHANNESSON, M. 1999. Probability weighting and utility curvature in QALY-based decision making. *Journal of mathematical psychology,* 43**,** 238-260.

DOCTOR, J. N., BLEICHRODT, H. & LIN, H. J. 2010. Health utility bias: a systematic review and meta-analytic evaluation. *Medical Decision Making,* 30**,** 58-67.

MARTIN, A. J., GLASZIOU, P., SIMES, R. & LUMLEY, T. 2000. A comparison of standard gamble, time trade-off, and adjusted time trade-off scores. *International Journal of Technology Assessment in Health Care,* 16**,** 137-147.

OLIVER, A. 2003. The internal consistency of the standard gamble: tests after adjusting for prospect theory. *Journal of health economics,* 22**,** 659-674.

PERPIÑÁN, J. M. A., MARTÍNEZ, F. I. S., PÉREZ, J. E. M. & MARTÍNEZ, I. M. 2009. Debiasing eq-5d tariffs. New estimations of the Spanish EQ-5D value set under nonexpected utility. Centro de Estudios Andaluces.

PINTO-PRADES, J.-L. & ABELLAN-PERPIÑAN, J.-M. 2012. When normative and descriptive diverge: how to bridge the difference. *Social Choice and Welfare,* 38**,** 569-584.

STIGGELBOUT, A. M., KIEBERT, G. M., KIEVIT, J., LEER, J.-W. H., STOTER, G. & DE HAES, J. 1994. Utility assessment in cancer patients: adjustment of time tradeoff scores for the utility of life years and comparison with standard gamble scores. *Medical Decision Making,* 14**,** 82-90.

TVERSKY, A. & KAHNEMAN, D. 1992. Advances in prospect theory: Cumulative representation of uncertainty. *Journal of risk and uncertainty,* 5**,** 297-323.

VAN DER POL, M. & ROUX, L. 2005. Time preference bias in time trade-off. *The European Journal of Health Economics,* 6**,** 107-111.

VAN OSCH, S. M., WAKKER, P. P., VAN DEN HOUT, W. B. & STIGGELBOUT, A. M. 2004. Correcting biases in standard gamble and time tradeoff utilities. *Med Decis Making,* 24**,** 511-7.

WAKKER, P. & DENEFFE, D. 1996. Eliciting von Neumann-Morgenstern utilities when probabilities are distorted or unknown. *Management science,* 42**,** 1131-1150.
